# Unraveling two decades of phyllosphere endophytes: tracing research trends and insights through visualized knowledge maps, with emphasis on microbial interactions as emerging frontiers

**DOI:** 10.1007/s44154-024-00148-y

**Published:** 2024-02-06

**Authors:** Muhammad Atif Muneer, Xiaohui Chen, Hexin Wang, Muhammad Zeeshan Munir, Muhammad Siddique Afridi, Xiaojun Yan, Baoming Ji, Wenqing Li, Liangquan Wu, Chaoyuan Zheng

**Affiliations:** 1 International Magnesium Institute, College of Resources and Environment, Fujian Agriculture and Forestry University, Fuzhou, 350002, China; 2 Anhui Province Key Lab of Farmland Ecological Conservation and Pollution Prevention; Anhui Province Engineering and Technology Research Center of Intelligent Manufacture and Efficient Utilization of Green Phosphorus Fertilizer, College of Resources and Environment, Anhui Agricultural University, Hefei, 230036 China; 3 School of Environment and Energy, Peking University Shenzhen Graduate School, 2199, Lishui Rd, Shenzhen, 518055 China; 4 Department of Plant Pathology, Federal University of Lavras (UFLA), Lavras, MG CEP 37200-900 Brazil; 5 College of Grassland Science, Beijing Forestry University, Beijing, China; 6 Fujian Institute of Tobacco Sciences, Fuzhou, 350013 China

**Keywords:** Phyllosphere, Endophyte, Review, Bibliometric analysis, CiteSpace, Microbial interaction

## Abstract

Phyllosphere endophytes play a critical role in a myriad of biological functions, such as maintaining plant health and overall fitness. They play a determinative role in crop yield and quality by regulating vital processes, such as leaf functionality and longevity, seed mass, apical growth, flowering, and fruit development. This study conducted a comprehensive bibliometric analysis aiming to review the prevailing research trajectories in phyllosphere endophytes and harness both primary areas of interest and emerging challenges. A total of 156 research articles on phyllosphere endophytes, published between 2002 and 2022, were retrieved from the Web of Science Core Collection (WoSCC). A systematic analysis was conducted using CiteSpace to visualize the evolution of publication frequency, the collaboration network, the co-citation network, and keywords co-occurrence. The findings indicated that initially, there were few publications on the topic of phyllosphere endophytes. However, from 2011 onwards, there was a notable increase in the number of publications on phyllosphere endophytes, gaining worldwide attention. Among authors, Arnold, A Elizabeth is widely recognized as a leading author in this research area. In terms of countries, the USA and China hold the highest rankings. As for institutional ranking, the University of Arizona is the most prevalent and leading institute in this particular subject. Collaborative efforts among the authors and institutions tend to be confined to small groups, and a large-scale collaborative network needs to be established. This study identified the influential journals, literature, and hot research topics. These findings also highlight the interconnected nature of key themes, e.g., phyllosphere endophyte research revolves around the four pillars: diversity, fungal endophytes, growth, and endophytic fungi. This study provides an in-depth perspective on phyllosphere endophytes studies, revealing the identification of biodiversity and microbial interaction of phyllosphere endophytes as the principal research frontiers. These analytical findings not only elucidate the recent trajectory of phyllosphere endophyte research but also provide invaluable insights for similar studies and their potential applications on a global scale.

## Introduction

Plant's microbiome is composed of diverse microorganisms living on its surface and within its tissues. Generally, plant microbiomes can be classified as phyllosphere, rhizosphere, and bulk soil microbiomes (Dastogeer et al. 2020). The phyllosphere, on the other hand, refers to the aboveground compartments of terrestrial plants, which include vegetative (leaves and stems) and reproductive (flowers, fruits, seeds) organs (Koskella 2020). In the phyllosphere, diverse microbes live in epiphytic (growing on the surface of a plant) and endophytic (residing within the plant) niches (Vorholt 2012; Zhu et al. 2022). Thus, phyllosphere endophytes are any microbes that inhabit internal tissues of aboveground compartments of plants without causing disease (Ramos et al. 2023). The phyllosphere is a unique habitat for diverse microbes, greatly affecting plant performance (Zhu et al. 2022). The phyllosphere, with a particular emphasis on the endophytic phyllosphere, plays critical roles in a myriad of biological functions, e.g., plant health and overall fitness, and determinative for crop yield and quality by influencing vital processes such as leaf functionality and longevity, seed mass, apical growth, flowering, and fruit development (Stone et al. 2018; Liu et al. 2020). Additionally, these endophytes play a substantial role in mitigating environmental contaminants (Thapa and Prasanna 2018). Nonetheless, to advance modern eco-friendly agricultural practices, it is imperative to gain a comprehensive understanding of the holistic ecology within plant–microbe associations, particularly for beneficial microbes such as endophytes. Such knowledge is integral to the sustainable management and enhancement of crop systems.

The phyllosphere, unlike the rhizosphere, is frequently subjected to high fluctuations in temperature, ultraviolet radiation, humidity, and nutrient availability, making it a harsher environment for microbiota (Spence and Bais 2013; Thapa and Prasanna 2018). Furthermore, the anthropocene can positively or negatively influence the assembly of microbes in the phyllosphere (Zhan et al. 2022). For example, scientists have observed a shift in the phyllosphere composition of tree leaves across a gradient of urbanization in Europe and North America, suggesting the phyllosphere microbiota is under pressure from the anthropocene (Laforest-Lapointe et al. 2017; Imperato et al. 2019). In the short term, anthropogenic activities, such as agrochemical applications, fertilizers, and nanotechnology, may contribute to crop fitness. Nevertheless, it remains to be determined whether their chronic effects on commensal microbiota are unintended (Perreault and Laforest-Lapointe 2022). Understanding the ways in which these cues influence microbial community assembly is a primary and essential step toward the extraction of functional microbial taxa from the phyllosphere. Hence, to gain a comprehensive grasp of these complex dynamics within this vital ecosystem, it is essential to understand the trends and characteristics of phyllosphere endophytes.

Globally, the phyllosphere represents an exciting area of research with considerable potential applications in ecology and related fields, but scholars around the world have paid relatively little attention to it. So far, various studies have been conducted on the phyllosphere (Perreault and Laforest-Lapointe 2022; Zhu et al. 2022); however, quantitative approaches are rarely used to generalize and summarize. The traditional method of literature analysis is useful for covering a large amount of information, but it has some obvious limitations, including a knowledge gap, a tendency to omit literature, and a lack of repeatability. In contrast to traditional qualitative methods, quantitative literature analysis can provide researchers with specific information more easily and quickly, e.g., bibliometric analysis. This method is useful for analyzing the characteristics and hot topics of a particular field, as well as identifying emerging trends in the field. Currently, there exists a lack of bibliometric analysis regarding the research hotspots and frontiers within the field of phyllosphere endophytes. The primary data source in this study is the Web of Science Core Collection Database (WoS), which is analyzed using bibliometric software such as CiteSpace and VOSviewer. It does statistical analysis on publication volumes, countries, publishing institutions, keyword clustering, and emerging keywords. In addition, it effectively organizes the background knowledge regarding phyllosphere endophytes. By analyzing these data, the study aims to identify the research focus, hotspots, and development trends of phyllosphere endophytes. The key objective is to provide valuable insights and references for researchers working on phyllosphere endophytes around the world, thereby contributing to the understanding and advancement of this emerging field.

## Results and discussion

### Changes in the number of published research articles

**Fig. 1:**
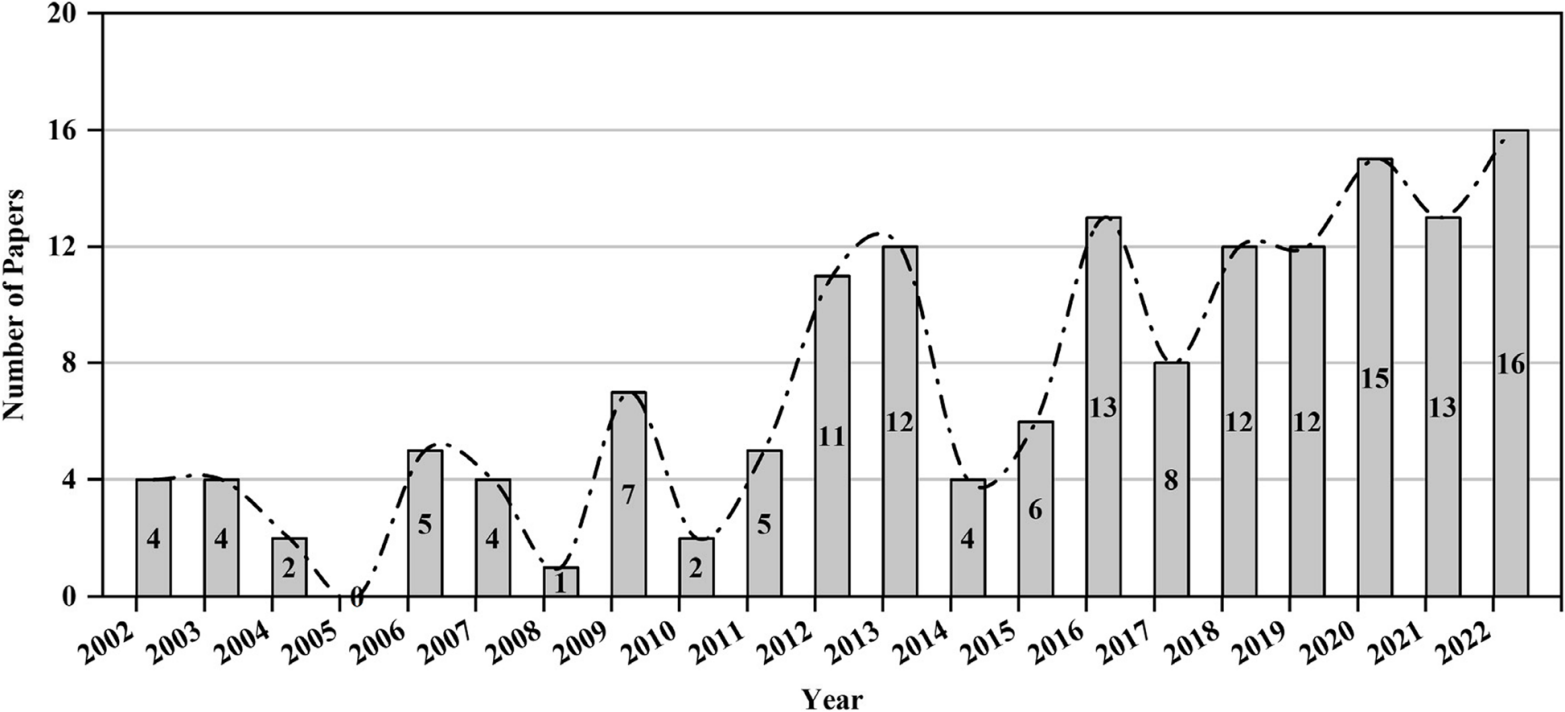
Number of papers per year in the field of phyllosphere endophytes in WoS during 2002–2022. The numbers on the bar represents the number of published papers during that specific year

The number of papers published each year reflects the dynamic change within the field of research. It provides insight into the depth of research and development trajectory of a specific field based on the number of publications in the field (Huang et al. 2020). The articles related to phyllosphere endophytes around the world from 2002 to 2022 showed modest research activity (Fig. 1). Hence, a few articles were published during this period, indicating a phase of low research attention. We found 34 publications from 2002 to 2011. It appears that during this time, the scientific community had only just begun exploring the potential of phyllosphere endophytes. In the start, the annual publications hardly exceeded five until 2011, except in 2009, where seven publications were recorded, highlighting the limited research attention of the researchers.

However, over time, after 2011, more researchers started to focus on the phyllosphere endophytes, gaining worldwide attention. As a result, the maximum number of publications were recorded during recent years of 2020 (15) and 2022 (16), showing international interest in the field of phyllosphere endophytes. There could be several reasons for this change in trajectory, including technological advancements, increased awareness of the ecological significance of phyllosphere endophytes, and pressing environmental challenges that prompted scientists to investigate new research avenues (Milazzo et al. 2021; Xu et al. 2022; Sohrabi et al. 2023). In the field of phyllosphere endophytes, global recognition and collaborative efforts have led to an increase in publications over the past few years. Researchers in multiple countries are collaborating and sharing their research outputs simultaneously, indicating a shared interest. As a result of this international interest, phyllosphere endophytes are gaining importance among scientists worldwide.

### Co-authorship analysis

Co-authorships analysis can provide a valuable way of visualizing and depicting collaboration patterns among researchers in a particular field of study. In this study, we analyzed the cooperation networks from the authors, countries, and research institutes' perspectives for a given field of study. It explores the research power and the intensity of collaboration among different nodes in the global research network.

#### Author co-authorship analysis

**Fig. 2:**
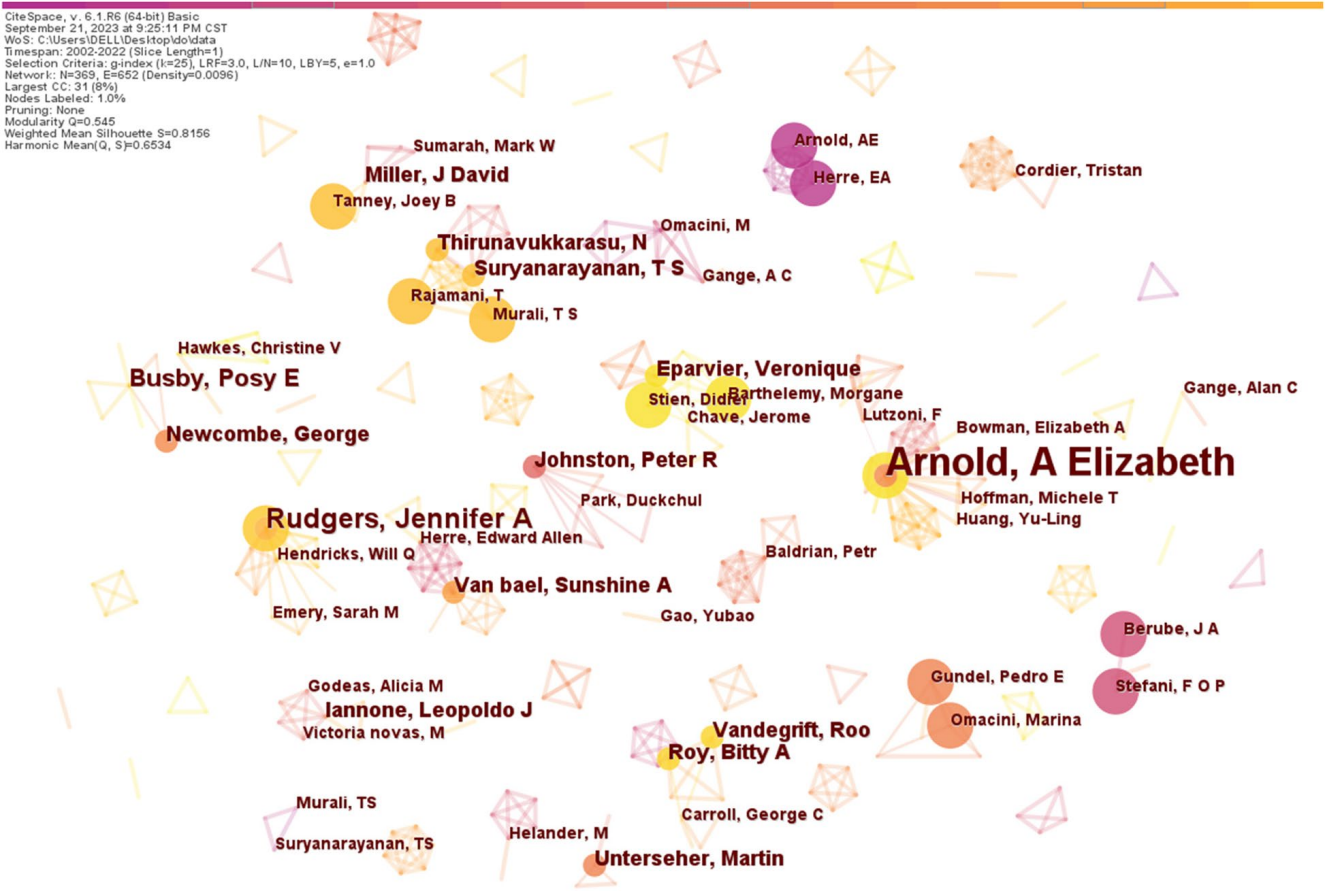
Map of the phyllosphere endophyte authors co-authorship network. The circle node size represents the number of published papers, and the connection between nodes reflects the cooperative relationship. The nodes in the network show the authors, larger the node, the more number of publications, whereas connecting lines (edges) represent the cooperative relationship between the authors. The color from light purple to light yellow indicates the change of time from early to recent time

It is possible to identify the core authors of the literature and their contribution to the research in the specific field by analyzing the authors of the literature and their collaborations, which promotes academic exchange and cooperation between researchers (Zheng et al. 2023). Authors' cooperation network mapping showed 369 nodes and 652 connecting lines (Fig. 2). The nodes in the network show the authors; the larger the node, the more number of publications, whereas connecting lines represent the cooperative relationship between the authors. Here, we found that among the top 10 authors, the top three authors by number of publications are Arnold, A Elizabeth (12), Rudgers, Jennifer A (5) and Busby, Posy E (4) (Table 1). The other authors among the top 10 also had a few publications, each with three publications (Table 1). This indicates that research focus on phyllosphere endophytes is still limited. We found that Arnold, A Elizabeth is more central and tends to have a greater capacity to influence others and a higher collaboration/co-authorship network as its centrality value is higher than that of other authors (Table 1). Hence, Arnold, A Elizabeth published the most articles related to the phyllosphere endophyte field in our analysis. She is one of the early researchers from the University of Arizona who studied phyllosphere endophytes, specifically the diversity and host range of foliar fungal endophytes (Arnold and Lutzoni 2007). The other scholars, like Rudgers, Jennifer A., Busby, and Posy E., have co-authored five and four articles, respectively. Their articles about plant endophytes also have received considerable attention(Rudgers et al. 2004; Busby et al. 2016). Overall, we found that the cooperation network is scattered and clustered into various small groups and disconnected clusters, which indicates the authors of the phyllosphere endophyte field were more likely to collaborate in small groups. Our findings suggest opportunities for enhanced communication and collaboration on a large scale.

**Table 1:** Top 10 authors in English literature published

Order number	Author	Number of publications	Centrality
1	Arnold, A Elizabeth	12	0.01
2	Rudgers, Jennifer A	5	0.00
3	Busby, Posy E	4	0.00
4	Suryanarayanan, T S	3	0.00
5	Van bael, Sunshine A	3	0.00
6	Unterseher, Martin	3	0.00
7	Newcombe, George	3	0.00
8	Thirunavukkarasu, N	3	0.00
9	Johnston, Peter R	3	0.00
10	Miller, J David	3	0.00

#### Country co-authorship analysis

**Fig. 3:**
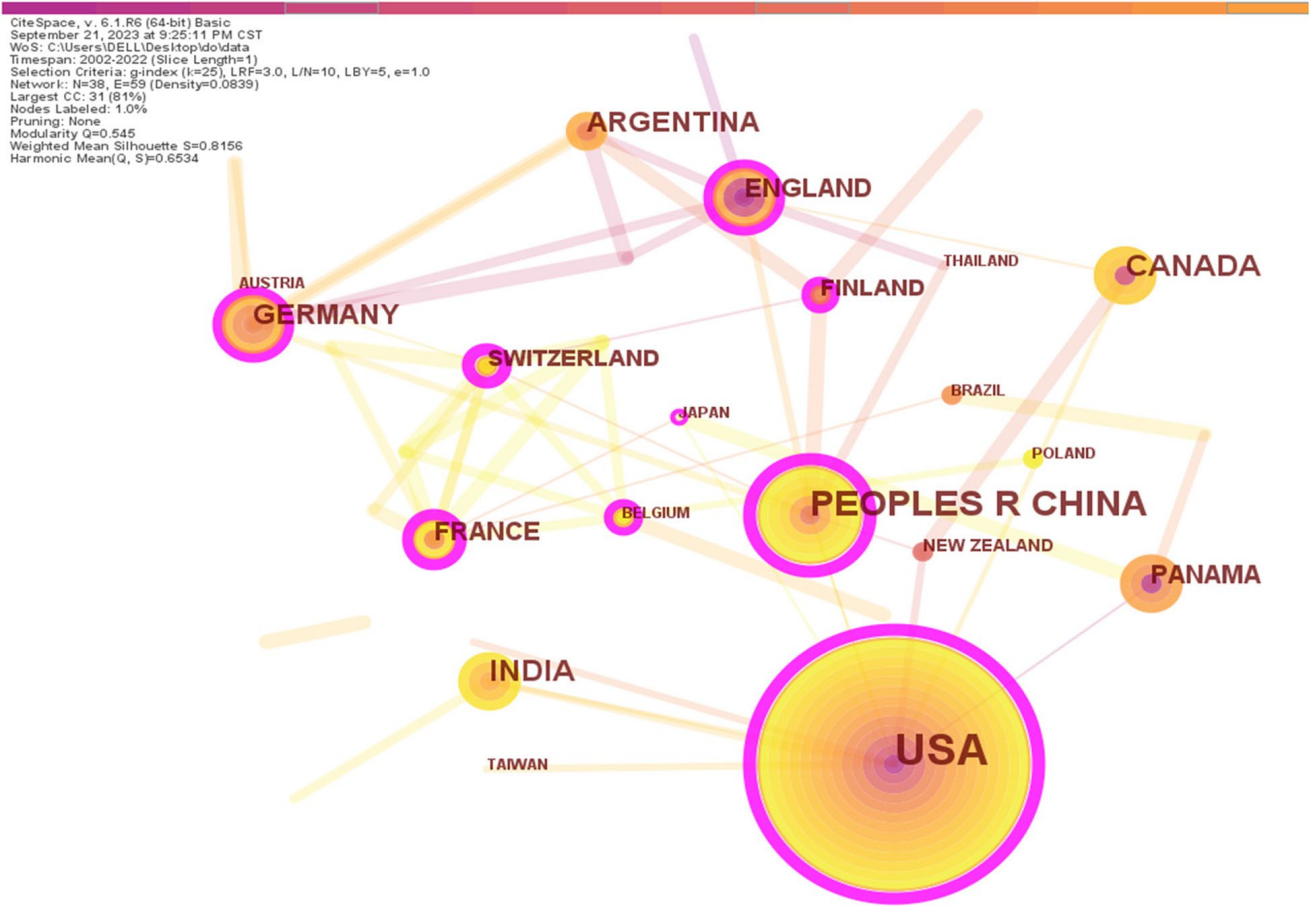
Visualization network map of the country co-authorship analysis. Across the map, each node represents a country. The circle node size represents the number of published papers, the purple outer ring represents the betweenness centrality, the ring color and thickness represent the year of occurrence, and the connection between nodes reflects the cooperative relationship

Changes in the country's published papers may represent a country or region's attention towards a specific field of research. Hence, a network map was illustrated to highlight the cooperation between different countries in the field of phyllosphere endophytes research during the time span of 2002–2022, with a total of 38 nodes and 59 edges or connections (Fig. 3). Across the map, each node represents a country, and the node size is proportional to the number of articles contributed by that country. The connections between nodes indicate cooperative relationships between countries. In the network, a purple outer circle represents mediation centrality, a metric that identifies the importance of intermediary nodes. In this process, literature sources are identified and quantified in order to determine their importance (Wang et al. 2016). For example, nodes that have an intermediary centrality greater than 0.1 are considered key nodes (Liu et al. 2019). However, national betweenness centrality is a measure of the international influence of a country in a given field of research (Sun et al. 2022). As a result, a sparse network of connections was apparent from the visual analysis, which indicated limited engagement of countries in the field, hence, an urgent need for improved international collaboration among countries.

The top 10 countries with the most papers published were selected and summarized in Table 2. The USA and China have top numbers of publications, which indicates these countries play substantial roles in the phyllosphere endophyte research field. The purple-rimmed nodes denote the countries with higher centrality values, which are recognized as junctions of the research network (Azam et al. 2021). The USA leads the list on top with 54 publications, accounting for 34.60% of the total number of papers in the WoS database. Hence, the USA has a significant contribution and has the highest value of centrality with 0.54, followed by China (21 publications with a centrality value of 0.51), Canada (15 publications with a centrality value of 0.05), India (13 publications with a centrality value of 0.09), Argentina (12 publications with centrality value 0.03), and Germany (12 publications with centrality value 0.12). Switzerland, despite the least number of papers (7), has a higher centrality value (0.46), demonstrating higher international cooperation with other countries. Although Canada's number of publications is more than two times that of Switzerland, its centrality value was still lower than that of Switzerland, meaning Canadian researchers have worked more independently than researchers from Switzerland. In short, the USA and China, with the highest centrality values, show their collaboration with other countries in the field related to phyllosphere endophyte. Consequently, fostering increased international exchanges and cooperation between different countries is essential to achieve breakthroughs and bolster global influence and awareness of phyllosphere endophytes research.

**Table 2:** Top 10 countries for the number of English publications

Order number	Country	Published paper	Centrality
1	USA	54	0.54
2	China	21	0.51
3	Canada	15	0.05
4	India	13	0.09
5	Argentina	12	0.03
6	Germany	12	0.12
7	Panama	8	0.06
8	France	8	0.25
9	England	8	0.17
10	Switzerland	7	0.46

#### Institution co-authorship analysis

**Fig. 4:**
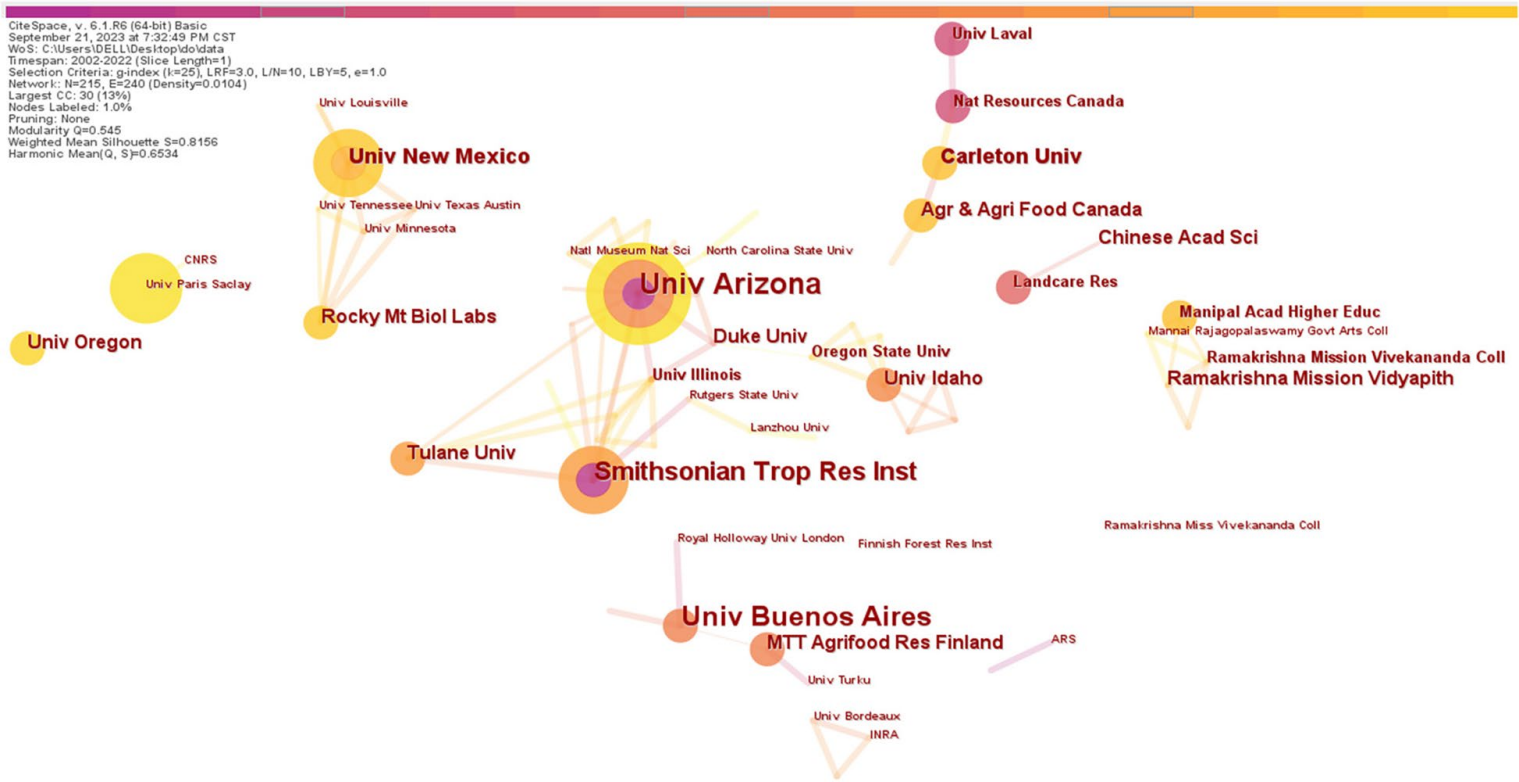
Visualization of the institution co-authorship network of phyllosphere endophyte. Each node represents the institute. The circle node size represents the number of published papers, the ring color and thickness represent the year of occurrence, and the connection between nodes reflects the cooperative relationship

The institutional collaborative relationship in the field of phyllosphere endophyte is mapped and visualized in Fig. 4. Like in country co-authorship analysis, each node represents the institute, and the node size is proportional to the number of articles contributed by that institute. Here, we found 215 nodes (institutes) and 240 edges in the institutional co-authorship network, and all the institutes are independent in the network. The detailed information on the top 10 institutes is shown in Table 3. The University of Arizona is the most frequently appearing and leading institute, with 13 papers among the top 10 institutes, while the centrality value is just 0.02, denoting relatively less influence of cooperation with other institutes. Meanwhile, the other institutes, like the University of Buenos Aires and Smithsonian Trop Res Inst with 10 and 09 papers, respectively, also showed a centrality value < 0.1, an indication of less influence and cooperation with other institutes, and overall work independently. Overall, these results show low collaboration at the institutional level. Hence, it is very important to focus on increasing collaboration with other research institutes to improve scientific research innovation in the field of phyllosphere endophytes. The research domain of phyllosphere endophytes can be better understood and innovated by fostering a culture of collaboration and removing institutional silos.

**Table 3:** Top 10 institutions in English literature published

Order number	Institution	Published paper	Centrality
1	University of Arizona	13	0.02
2	University of Buenos Aires	10	0.01
3	Smithsonian Trop Res Inst	9	0.01
4	Carleton University	6	0.00
5	New Mexico State University	6	0.00
6	University of Idaho	5	0.01
7	Duke University	4	0.01
8	Rocky Mountain Biological Laboratory	4	0.00
9	Ramakrishna Mission Vidyapith	4	0.00
10	Agriculture and Agri-Food Canada	4	0.00

### Co-citation analysis

Co-authorship analysis can provide insights for understanding the influence of research power on the phyllosphere endophyte research field. Nonetheless, it remains challenging to precisely depict the contribution of authors, journals, and literature contribution to the field of study. A co-citation relationship is defined as the occurrence of two or more authors, journals, or literature being cited together in a third document (Fang et al. 2018). In this part, two main types of co-citation analyses were used to identify the relationship and mapping structures of journals and literature.

#### Journal co-citation analysis

**Fig. 5:**
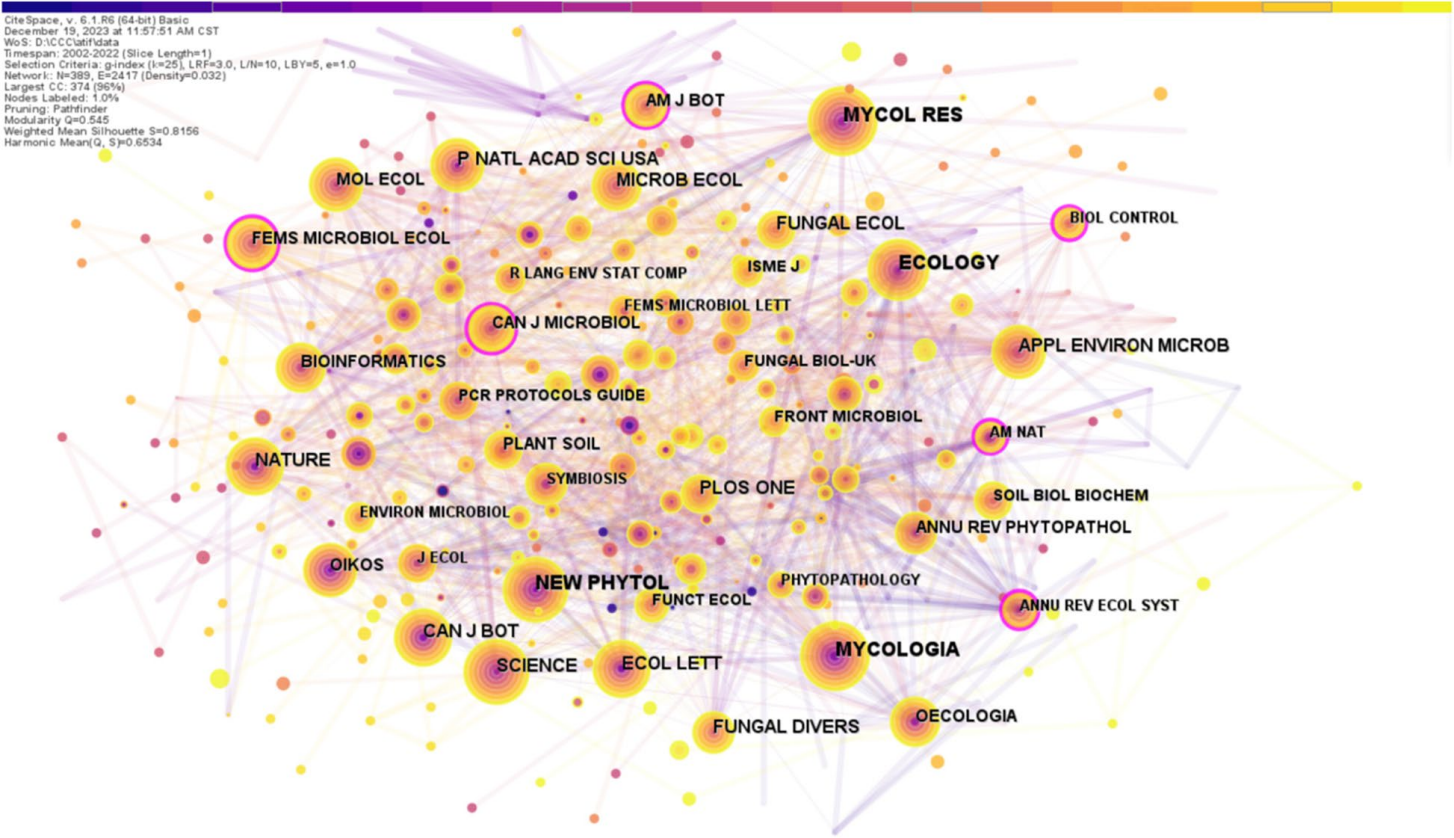
Map of the journal co-citation network. Each node represents a journal; the larger the node, the more times it is referenced. and the connecting lines between the nodes represents the co-citation relationship, and its thickness represents the co-citation strength. The color from purple dark to light yellow indicates the change of time from early to recent time

Identifying prominent journals within a field that publish academic research findings and disseminate scholarly information relies heavily on journal co-citation analysis (Azam et al. 2021). In addition to providing valuable references, this analysis assists researchers in conducting more efficient literature searches, and the number of citations denotes the paper impact on the research field (Wang et al. 2021). The co-citation network of the journal was analyzed to visualize the distribution of phyllosphere endophyte journals using CiteSpace (Fig. 5). The co-citation network resulted in 389 nodes and 2417 links. The size of each node represents the number of co-citations of the journal, and a larger node size means the journal has more influence. The top 10 most influential journals related to phyllosphere endophyte have been ranked based on co-citation frequency in Table 4. Among the top 10 journals, New Phytologist stands out with 108 co-citations, followed by Mycologia (106) and Mycological Research (103). As phyllosphere endophyte repositories, these journals are renowned for their high co-citation frequencies. As a result of these extensive citations, these journals are not only indicative of their depth of scholarly activity but have also played a significant role in shaping discourse and progress in the field of phyllosphere endophytes. Hence, the higher citation counts of the journals show that the most influential research is published in these journals.

**Table 4:** Top 10 most cited journals with co-citation frequency on phyllosphere endophyte

Order	Journal	Frequency
1	New Phytologist	108
2	Mycologia	106
3	Mycological Research	103
4	Ecology	96
5	Proceedings of the National Academy of Sciences	85
6	Plos One	77
7	Nature	73
8	Science	71
9	Fungal Ecology	70
10	Canadian Journal of Botany	65

#### Literature co-citation analysis

**Fig. 6:**
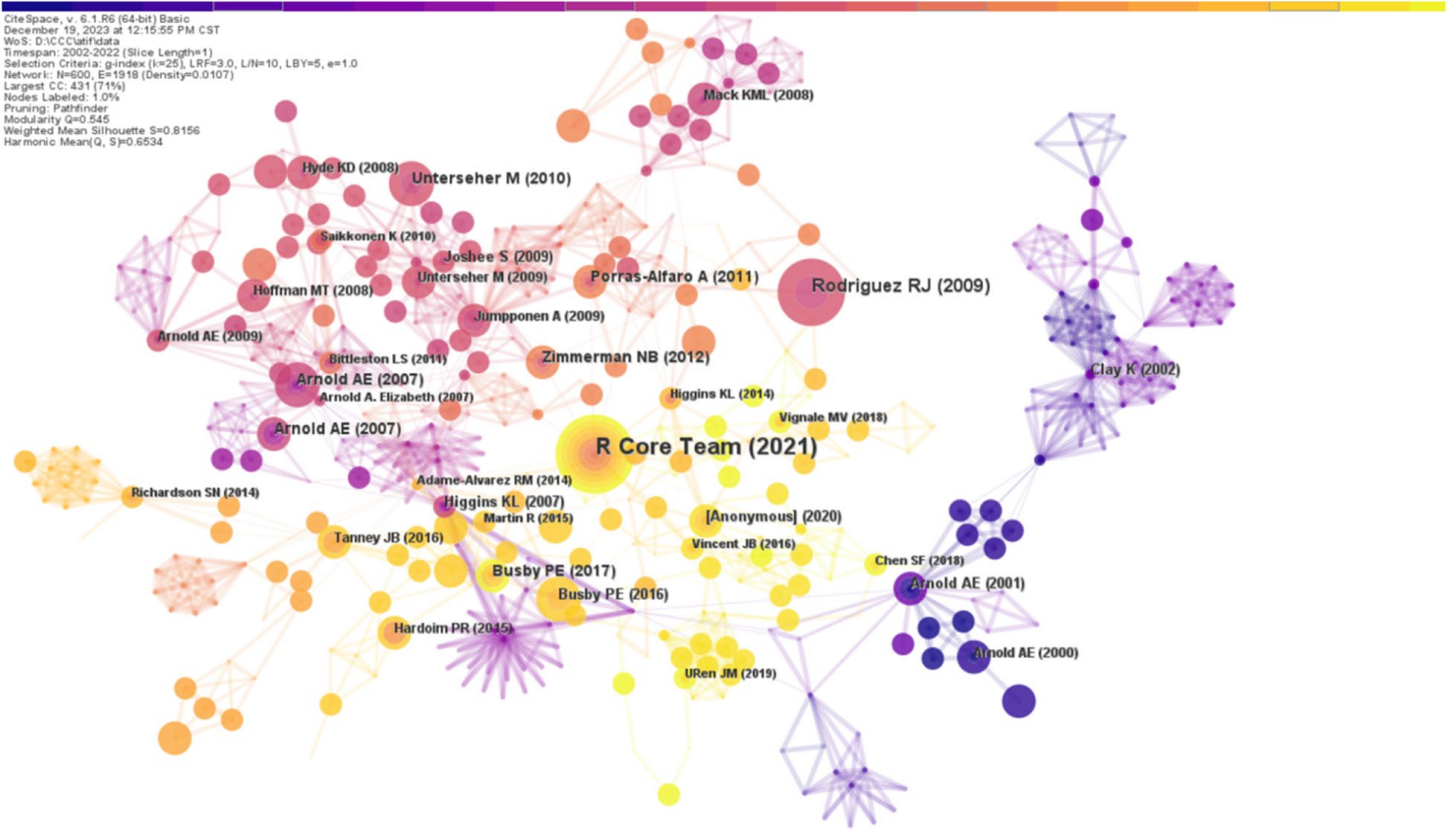
Visualization of the literature co-citation network. Each node represents a piece of literature, with larger nodes indicating a higher number of co-citations. The lines between the nodes represents the co-citation relationship, and its thickness represents the co-citation strength. The color from purple dark to light yellow indicates the change of time from early to recent time

The literature/document or article co-citation analysis is a valuable tool for uncovering research themes and tracing the research development in the specific research field. It also provides insights into the academic reputation of the field and its representative research expertise. The articles with significant co-citations are the core references in the phyllosphere endophyte field. The literature co-citation analysis was performed for phyllosphere endophyte studies. The visualization network is shown in Fig. 6. The network mapping of literature co-citation analysis showed 600 nodes with 1918 links and a network density of 0.0107, where each node represents an individual piece of literature, while the thickness of the edge represents the strength of co-citation between documents (Fig. 6). A larger node size signifies the document importance. In contrast, closely linked nodes depict that documents have a high co-citation frequency.

The top 10 highly co-cited articles have been shown in Table 5, where the article entitled "Fungal endophytes: diversity and functional roles" by Rodriguez RJ et al. (2009) is the most co-cited in our analysis with 12 co-citations. This article categorizes the endophytes according to their ability to colonize and transmit hosts, contribute to a plant's diversity, and benefit the host (Rodriguez et al. 2009). As a result of this categorization, we are able to understand the diverse roles that endophytes play in plant ecosystems, thereby guiding our subsequent research.

**Table 5:** Top 10 most cited references

Order	Title	Journal	Author	Frequency	Year
1	Fungal endophytes: diversity and functional roles	New Phytologist	Rodriguez RJ	12	2009
2	Diversity and Host Range of Fuliar Fungal Endophytes:Are Tropical Leaves Biodiversity Hotspote?	Ecology	Arnold AE	8	2007
3	Fungal endophyte communities reflect environmental structuring across a Hawaiian landscape	P NATL ACAD SCI USA	Zimmerman NB	7	2012
4	Hidden Fungi, Emergent Properties: Endophytes and Microbiomes	Annual Review of Phytopathology	Porras-Alfaro A	7	2011
5	Diversity and phylogenetic affinities of foliar fungal endophytes in loblolly pine inferred by culturing and environmental PCR	Mycologia	Arnold AE	7	2007
6	Research priorities for harnessing plant microbiomes in sustainable agriculture	PLOS Biology	Busby PE	7	2017
7	Species richness analysis and ITS rDNA phylogeny revealed the majority of cultivable foliar endophytes from beech (Fagus sylvatica)	Fungal Ecology	Unterseher M	7	2010
8	Evolutionary Origins and Ecological Consequences of Endophyte Symbiosis with Grasses	American Naturalist	Clay K	6	2002
9	Diversity and distribution of fungal foliar endophytes in New Zealand Podocarpaceae	Mycological Research	Joshee S	6	2009
10	Phylogenetic relationships, host affinity, and geographic structure of boreal and arctic endophytes from three major plant lineages	Molecular Phylogenetics and Evolution	Higgins KL	6	2017

A second highly co-cited article, "Diversity and host range of foliar fungal endophytes: Are tropical leaves biodiversity hotspots?" illustrates the importance of endophytes across diverse ecosystems. In this study, researchers highlighted the increase in endophyte incidence, diversity, and host range from arctic to tropical environments, emphasizing the need to take into consideration varied ecological contexts in the study of endophytes (Arnold and Lutzoni 2007). By analyzing literature co-citations, we can identify pivotal works and illuminate the key concepts and themes that have shaped the phyllosphere endophyte research landscape. Through these insights, researchers can navigate the vast body of literature, identifying important works and conceptual frameworks that continue to influence the field. Furthermore, the analysis provides scholars with an invaluable resource for exploring the historical evolution of ideas, anticipating future research directions, and contributing meaningfully to the ongoing discussion about phyllosphere endophytes.

### Hot research topics

#### Keywords co-occurrence analysis

The term "hot research topics" refers to areas of study that are currently receiving the most attention and investment from the scientific community and funding agencies (Li et al. 2023). Hence, keywords are typically used to summarize the core content of an article, reflecting the article's value, objectives and methods (Li et al. 2019). Keyword co-occurrence analysis could serve as a robust technique for investigating the most influential research literature on phyllosphere endophytes by examining the frequency and betweenness centralities of co-occurring keywords (Chen and Liu 2020). Hence, to unveil the research hotspots of the phyllosphere endophyte field, keywords co-occurrence network analysis was executed.

**Fig. 7:**
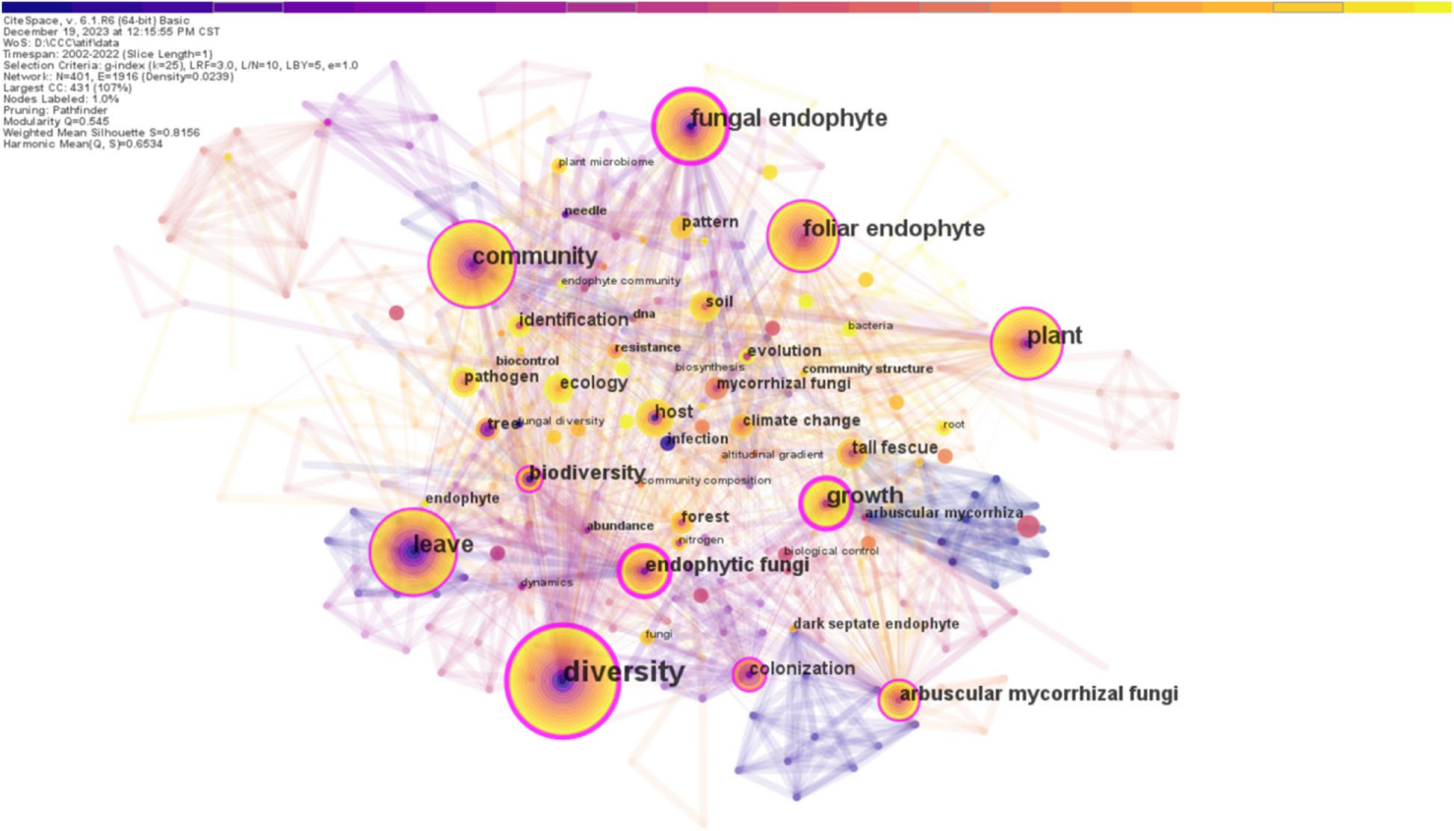
Visualization of keyword co-occurrence network map. Each node in the network represent a keyword. The link between the nodes represents the co-occurrence between keywords. The size of the circle means the rate of occurrence of the keywords. The color from purple dark to light yellow indicates the change of time from early to recent time

Keywords co-occurrence network was constructed via CiteSpace (Fig. 7) based on 156 research papers on the phyllosphere endophyte domain and obtained 401 nodes and 1916 edges. The size of each circle reflects the frequency of keyword occurrences in the literature, effectively showing hotspots of research in the field. In order to determine the frequency of co-occurrence within a document, connections between nodes are thicker when the nodes are strongly associated with each other (Fig. 7). Top 10 keywords were ranked by frequency (Table 6). The top five keywords with higher frequency are Diversity (65 occurrences), Plant (33 occurrences), Fungal endophyte (33 occurrences), Leave (33 occurrences), and Community (32 occurrences). In terms of centrality, 'Diversity' (0.31), 'Fungal endophyte' (0.26), 'Growth' (0.22) and 'Endophytic fungi' (0.21) are more prominent, suggesting their importance and their key role in highlighting current research hotspots within this field. It is noteworthy that these keywords emerged as the most frequently occurring terms, underlining their significance in the current research discourse. The keywords express the importance of understanding endophyte diversity, how it interacts with hosts, and how it plays a role in the ecological communities of phyllosphere endophytes.

**Table 6:** Keywords of the top 10 frequency in the study

Order number	Frequency	Centrality	Year	Keyword
1	65	0.31	2002	Diversity
2	33	0.13	2004	Plant
3	33	0.26	2002	Fungal endophyte
4	33	0.11	2002	Leave
5	32	0.19	2003	Community
6	27	0.13	2002	Foliar endophyte
7	24	0.22	2002	Growth
8	17	0.19	2003	Arbuscular mycorrhizal fungi
9	17	0.15	2002	Biodiversity
10	17	0.21	2003	Endophytic fungi

#### Keywords clustering analysis

**Fig. 8:**
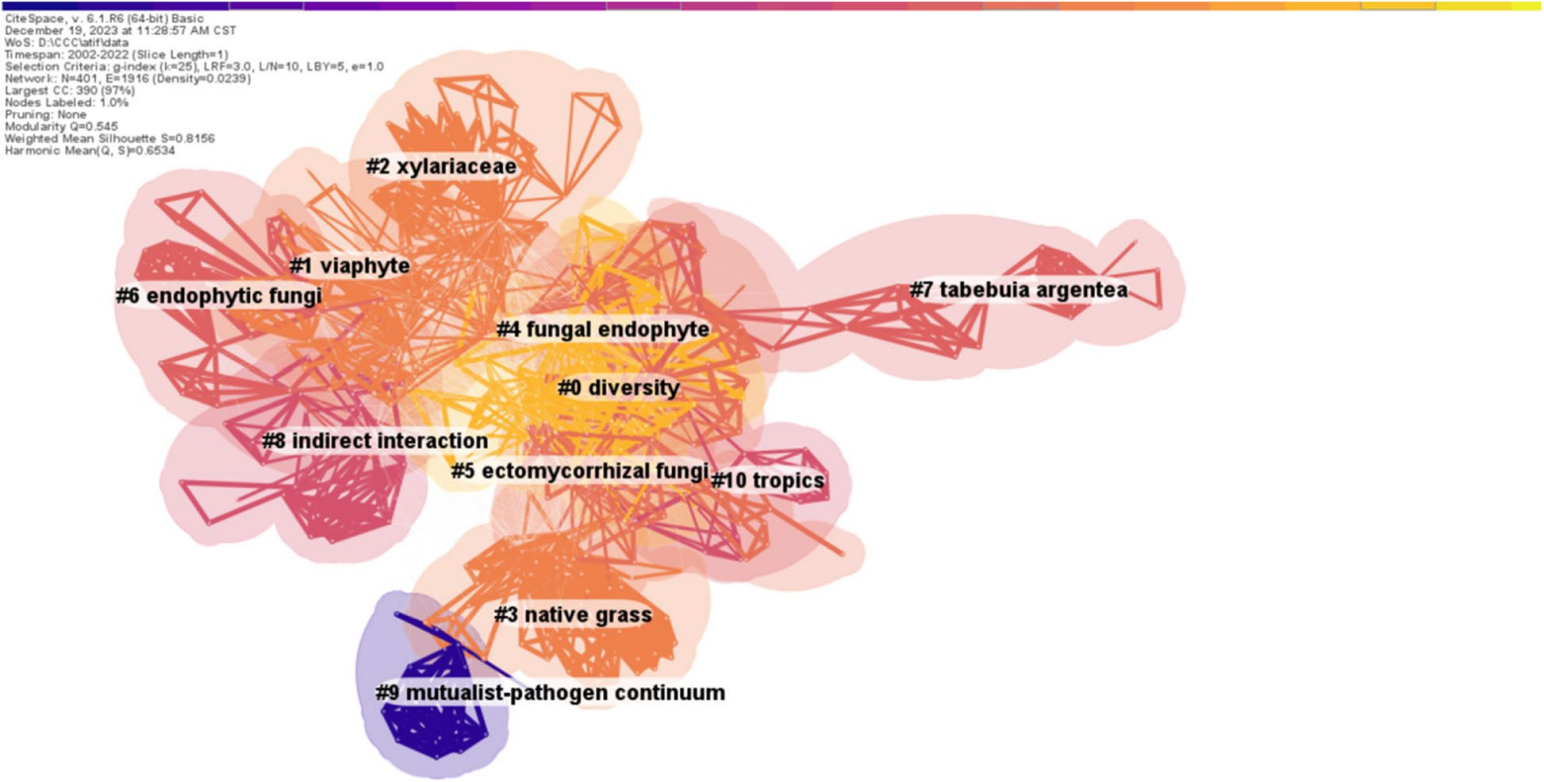
Keyword co-citation cluster network. The clustering criterion is guided by two indicators: a clustering modularity value (Q) exceeding 0.3 signifies significant clustering results, with larger values indicating better outcomes; moreover, a clustering profile index (S) surpassing 0.5 indicates reasonable clustering results. The values of Q and S were 0.545 and 0.8156, respectively, affirming the ideal quality of the clustering results. Different colors represent different clusters; the smaller the number, the more keywords are included in the cluster. The color from purple dark to light yellow indicates the change of time from early to recent time

A keyword clustering analysis was performed to analyze current trends and topics in phyllosphere endophyte. The cluster analysis of keywords has been essential for categorizing keywords, grouping those with a high degree of similarity, and revealing the phyllosphere endophytes research thematic structure. In this study, we find that the network map had 401 nodes and 1916 connections (Fig. 8), and organized the keywords into 11 clusters (Table 7), namely: #0 "diversity", #1 "viaphyte", #2 "xylariaceae" #3 "native grass", #4 "fungal endophyte", #5 "ectomycorrhizal fungi", #6 "endophytic fungi, #7 "tabebuia argentea", #8 "indirect interaction", #9 "mutualist-pathogen continuum", # 10 "tropics". Hence, based on keywords co-occurrence network cluster analysis, the research domain of phyllosphere can be summarized into the following key aspects:

**Table 7:** Top 11 clusters label information

Cluster ID	Cluster size	Contour value	Mean year	Cluster keywords (Top Three)
#0	59	0.633	2012	Diversity; Identification; Plant
#1	56	0.816	2013	Viaphyte; Shannon diversity; Cronartium ribicola
#2	45	0.858	2012	Xylariaceae; populus; Plant microbiome; Colonization
#3	44	0.794	2009	Native grass; Arbuscular mycorrhiza; Bromus auleticus
#4	34	0.808	2011	Fungal endophyte; Phthalides; Grasses
#5	33	0.800	2015	Ectomycorrhizal fungi; Foliar endophyte; Foliar endophytes
#6	31	0.868	2010	Endophytic fungi; Barro colorado island; Edenia gomezpompae
#7	29	0.946	2013	Tabebuia argentea; Persistence; Secondary metabolite
#8	25	0.860	2010	Indirect interaction; Community genetics; Burkholderia
#9	14	0.936	2005	Mutualist-pathogen continuum; Peppermint; Secondary metabolism
#10	13	0.912	2012	Tropics; Colonization frequency; Colletotrichum dematium

Biodiversity and identification of phyllosphere endophytes (Cluster #0, #1). This cluster signifies the importance of understanding the diversity and community composition of the microbiota, such as bacteria and fungi inhabiting the phyllosphere, the aboveground part of plants, including leaves, fruits, and stems. Scientists are likely looking at the various strains and species of endophytic microbes present in the phyllosphere. The identification and characterization of these microorganisms is essential to understanding their potential benefits or harm to plants, as well as their roles in ecosystem processes. For example, the bacterial community is considered the predominant group of phyllosphere microbiota, with an average ranging from 10^6^–10^8^ cells cm^2^ of leaf tissue, constituting about 10^26^ bacterial cells globally (Bashir et al. 2022). Plant phyllosphere is characterized by Proteobacteria, Bacteroides, Firmicutes, and Actinobacteria, the four main groups of bacteria (Carvalho et al. 2020). Proteobacteria possess a variety of metabolic capabilities, including N-fixation, nitrification, methylotrophy, and anoxygenic photosynthesis (Watanabe et al. 2016; Muneer et al. 2022; Zheng et al. 2022). Bacteroidetes found in the leaf often include aerobic and pigmented bacteria (Bashir et al. 2022). Firmicutes and Actinobacteria are often found in arid environments; thus, they are capable of surviving harsh conditions (Stankovic et al. 2012; Mina et al. 2020). Furthermore, the phyllosphere also supports a large diversity of fungi but is less diverse than bacteria because fungal communities are more sensitive to environmental variations (Chen et al. 2022). The fungal community also plays a vital role as a key component of the leaf microbiome, significantly affecting the host plant in various ways (Bashir et al. 2022). For example, the decomposition of leaf-litter and recycling of various nutrients, specifically nitrogen and carbon (Guerreiro et al. 2018), sustainability of ecosystem productivity (Van Der Heijden et al. 2016), biotic and abiotic stress tolerance (Arnold et al. 2007; Guerreiro et al. 2018). Recent advancements in technology, such as next-generation sequencing, proteogenomics, and metaproteogenomics, have enabled us to analyze microbial communities in new ways. These approaches allow us to study how these communities are formed and to identify potential areas for further research.

Microbial Interactions (Cluster #2, #3, #4, #5, #6, #7, #8, #9, #10): Microbial interactions play a vital role in maintaining ecological balance. These interactions enable the microbiota to thrive under diverse conditions and facilitate crucial processes, especially the interaction between plants and microorganisms, which play a fundamental role in natural ecosystems (Kumar et al. 2016; de Medeiros et al. 2021). Plant-microorganism interactions occur at numerous stages of plant development, from germination to senescence, and encompass a variety of relationships from protective habitats to nutrient-rich environments. In this interaction, plants provide a sheltered environment for microbes and produce organic and inorganic compounds that provide nutrients, encouraging microbial colonization. Conversely, microbes can influence plant physiology through pathogenic, commensal, mutualistic, or amensalistic interactions, which highlight highlighting the complexity of these relationships (Kumar et al. 2016; Schirawski and Perlin 2018). Hence, plant–microbe interactions play crucial roles in shaping plant health and growth. These interactions involve a wide array of microorganisms, including bacteria, fungi, and viruses, engaging with plants in various ways. The plant typically hosts these multitudes of microbial communities in different compartments, for instance, in the phyllosphere and rhizosphere; hence, they influence the host health and physiology through various trajectories (Hamonts et al. 2018; Trivedi et al. 2020).

Phyllosphere endophytes are microorganisms that live on the aboveground sections of plants and form a dynamic and diversified ecological community. These endophytes colonize the internal organs of the plant without causing visible symptoms. The interaction between plants and their associated phyllosphere microbial communities has received increasing attention during the last decade (Hacquard and Schadt 2015). The endophytes consist of a diverse range of bacteria, fungi, and other microorganisms that establish complex interactions with their host plants (Afridi et al. 2022b). For example, interactions between plants and phyllosphere endophytes have been demonstrated to enhance plant growth and bolster host resilience against biotic and abiotic stresses. In numerous instances, endophytic microbes have been documented to play an important role in plant stress protection and development. Various mechanisms, including hyperparasitism, competitive interactions, and antibiosis, can restrain the activity of plant pathogens. Fungal endophytes stimulate the production of phenolic compounds in perennial ryegrass, thereby enhancing the resistance against pathogenic growth (Pańka et al. 2013).

In the phyllosphere, the leaf harbors a substantial population of microorganisms, and the leaf endophytes have been found to affect the host fitness and growth (Davison 1988; Schauer and Kutschera 2011), enhance the resilience to environmental stresses (Vorholt 2012), and strengthen the resistance against pathogens (Innerebner et al. 2011). Leaf microbial communities originate from diverse sources, as microbes can colonize plant leaves vertically through seeds or pollen and horizontally from the air, soil, and insects (Frank et al. 2017; Chaudhry et al. 2021). Leaf microbial communities impact plant fitness by regulating the host plant's immune system and fostering growth in aboveground tissues (Singh et al. 2018). Several studies have found a relationship between leaf endophytic filamentous fungi and yeast with the host plants (Into et al. 2020; Chaudhry et al. 2021). Leaf endophytes were found to be highly diverse, spatially structured, and associated with host plants. They played an important role in protecting plants against the devastating foliar oomycete pathogen, Phytophthora sp. (Arnold et al. 2003). Moreover, pathogens and endophytes interact directly. It has been found that fungus endophytes in oak trees might be antagonistic to Erysiphe alphitoides, the causative agent of powdery mildew (Jakuschkin et al. 2016). Moreover, it has been reported that endophytes contain biosynthetic gene clusters, including non-ribosomal peptide synthetase (NRPS) and polyketide synthetase (PKS) genes (Miller et al. 2012; Ludlow et al. 2019), which may be useful for biocontrol.

So far, phyllosphere endophytes have gained substantial interest in the past few years due to their crucial influence on various ecosystems. Gaining insight into their functional responsibilities opened up possibilities for inventive applications in several fields, such as agriculture, biotechnology, and environmental sciences. For instance, agriculture stands to benefit immensely from phyllosphere endophyte research. These microbes have a vital function in promoting plant growth, facilitating nutrient absorption, and improving resistance to stress (Afridi et al. 2022a; Bashir et al. 2022). For example, some strains have shown the capacity to enhance crop productivity by assisting in the absorption of nutrients and reducing the negative effects of environmental stresses (Rana et al. 2020). The application of phyllosphere endophytes as biofertilizers and biopesticides offers a sustainable and eco-friendly approach to modern agriculture (Bashir et al. 2021). Likewise, its perspective in biotechnology shows great potential for the development of novel biocontrol agents to control pathogens and disease-causing insect pests. Moreover, phyllosphere-associated microbial communities produce compounds, such as indole acetic acid, gibberellic acids and cytokines that could play a key role in plant growth (Sivakumar et al. 2020). Hence, these microorganisms exhibit unique biochemical pathways and metabolic activities that can be harnessed for the production of bioactive compounds with pharmaceutical, industrial, or therapeutic applications. Uncovering the genetic capabilities of phyllosphere endophytes is crucial for harnessing a valuable array of biological resources. Phyllosphere endophytes play a significant role in maintaining the health and resilience of ecosystems in the field of environmental studies. Their interactions with host plants impact the process of nutrient cycling, the storage of carbon, and the overall functioning of the ecosystem (Vorholt 2012). Studying these microorganisms provides valuable insights into the intricate relationships between plants and their microbial inhabitants, with implications for biodiversity conservation and ecosystem restoration efforts (Bashir et al. 2022). A notable example of phyllosphere endophyte research in action is the identification of endophytic bacteria capable of degrading environmental pollutants. Researchers have made a significant discovery in a study where they found a type of phyllosphere endophyte that can degrade persistent organic contaminants. This finding suggests a possible approach for using living organisms to clean up polluted areas, known as bioremediation. In a groundbreaking study, researchers discovered a strain of phyllosphere endophyte with the ability to break down persistent organic pollutants, offering a potential bioremediation strategy for contaminated sites. This underscores the transformative potential of phyllosphere endophyte research in addressing pressing environmental challenges (Afzal et al. 2014; Zainab et al. 2020; Singh et al. 2022).

#### Keywords burst analysis

**Fig. 9:**
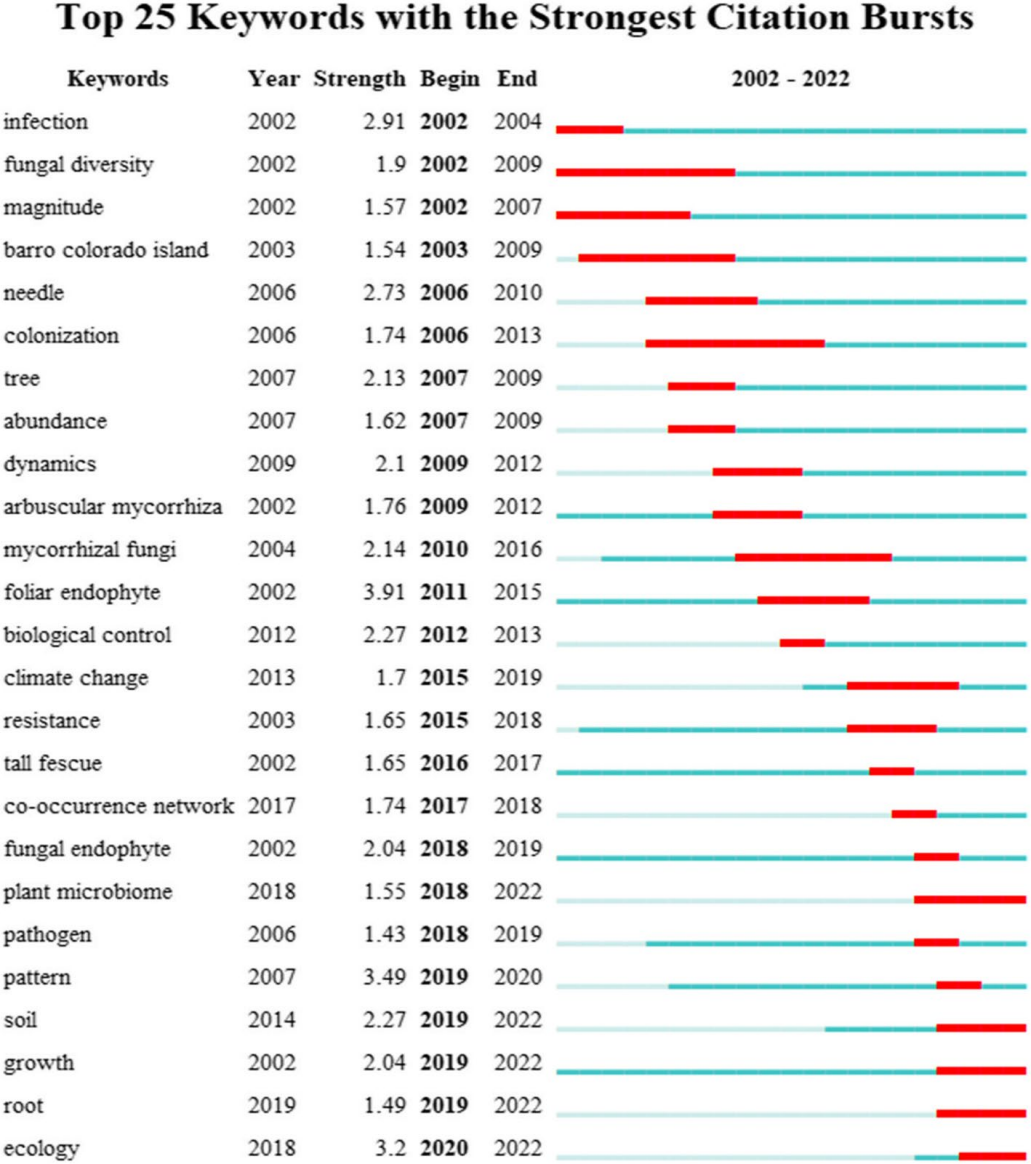
Top 25 keywords with the strongest citation bursts related to phyllosphere endophytes research published from 2002 to 2022. Year represents when keywords start appearing. Strength denotes the highlighting grade. Begin and End represent the starting and ending years of the keywords. The peacock green and red band indicate the time during which the keyword continues to appear and suddenly burst into citations, respectively

By analyzing the keyword burst (the keywords with a large change in frequency within a short time) in a large number of literature, CiteSpace could clearly show the research fronts/research hotspots in the field (Huang et al. 2023). Hence, nodes with high-frequency keywords are considered cutting-edge content within their field. It is important to recognize emerging directions and development trends within this field by analyzing these research frontiers. The burst strength and burst period are the leading indicators of burst detection. Based on keywords burst analysis, the top 25 keywords were obtained in the field of phyllosphere endophytes (Fig. 9). The red lines indicate when the burst words appeared and ended, and the strength shows the degree of influence. The greatest growing intensity was associated with the keyword 'foliar endophyte, ' reporting strength of 3.91, followed by 'pattern' and 'ecology,' with emergent strength of 3.49 and 3.20, respectively. This indicates that research on phyllosphere endophytes gained substantial attention from the scientific community from 2011 to 2022 and developed as a prominent research hotspot. Among these keywords, the most recent burst words were plant microbiome, soil, growth, root, and ecology, indicating this persistence will continue. Hence, keyword burst analyses provide valuable insight into current and emerging trends in phyllosphere endophyte research as well as focal points within the scientific community. This study identified areas of rapid development and increasing attention by identifying keywords with significant bursts in frequency over a defined period. It also highlights the interdisciplinary nature of contemporary research on phyllosphere endophytes by identifying current research hotspots. For example, the concepts of 'plant microbiome' and 'soil' emphasize how researchers are exploring endophytes' relationship with their environment holistically (Yadav 2020; Ayilara et al. 2023). Hence, to judge these keywords (plant microbiome, soil, growth, root, and ecology) is the current research frontier. These trends will likely shape the future of research, encouraging collaborative research that integrates diverse disciplines such as microbiology, ecology, and plant biology (Hardoim et al. 2015; Saikkonen et al. 2015; Liu et al. 2020; Muneer et al. 2021; Sohrabi et al. 2023). Nevertheless, researchers can strengthen their understanding of phyllosphere endophytes and their crucial role in ecosystem dynamics and plant health by staying attuned to these emerging trends.

## Conclusions

In this study, we have used CiteSpace for an in-depth analysis of 156 relevant publications with the key objective of visualizing the research evolution and landscape of phyllosphere endophytes. The findings of this analysis can be used to draw several conclusions.

Firstly, it is worth mentioning that the number of relevant studies relating to the topic of phyllosphere endophytes has substantially increased since 2012. Over time, the number of articles on this subject has consistently increased, attracting significant interest from a diverse range of fields, including multidisciplinary and cross-disciplinary areas. Scholars from the United States and China have shown a keen interest in phyllosphere endophytes research. Moreover, collaborative efforts among authors and institutions in this field typically cluster within small groups, indicating a need to establish a widespread collaborative network. Secondly, it is noteworthy that the most influential journal in this area of study of phyllosphere endophyte is undoubtedly New Phytologist. The most prominent and influential authors in this field include Arnold, A Elizabeth, Rudgers, Jennifer A, and Busby, Posy E. The most influential institutions in this field are predominantly located in the United States, such as the University of Arizona, New Mexico State University, University of Idaho, Duke University, and Rocky Mountain Biological Laboratory. Finally, in terms of current hot research topics, biodiversity and identification and microbial interaction of phyllosphere endophytes are the principal research frontiers in this study of phyllosphere endophytes. Besides highlighting the current research focus, these findings also highlight the interconnected nature of key themes. Phyllosphere endophyte research revolves around the four pillars: diversity, fungal endophytes, growth, and endophytic fungi. By gaining a better understanding of these focal points, researchers can guide their inquiries as well as inform funding agencies and policymakers about critical areas that need attention. Scientists could likely explore these themes in-depth in the future, advancing our understanding of how endophytes interact within the phyllosphere.

In the future, scholars and institutions should prioritize global collaboration in phyllosphere endophyte research. It is important to encourage international partnerships in order to establish strong networks, which will facilitate the exchange of knowledge and resources. In addition, interdisciplinary approaches are essential for exploring the practical applications of phyllosphere endophyte research in biotechnology, agriculture, and environmental sciences. In order to gain valuable insight for emerging researchers and institutions seeking to increase visibility, researchers should analyze influential factors like journals, authors, and institutions in depth. Moreover, cutting-edge technologies such as genomics and metagenomics must be integrated into the analysis. We can gain a greater understanding of microbial diversity and interactions within the phyllosphere by employing these technologies. Phyllosphere microbiome data can be analyzed with advanced tools and computational models, allowing scientists to develop innovative applications and sustainable solutions for agriculture, ecology, and biotechnology.

A limitation of this study is that we only included publications in the English language, which may lead to the loss of some articles. Hence, further investigations could consider analyzing and evaluating the literature on phyllosphere endophytes from different language databases for a better understanding of the global knowledge structure in the field of phyllosphere endophytes.

## Materials and methods

### Data extraction

The data used in this study were obtained from the WoS core collection database, a widely known source for bibliometric analysis. Make sure the retrieved data is comprehensive and accurate by using the 'Basic Searches' mode and setting the 'Topic' search as follows: "phyllosphere endophytes" OR "leaf endophytes" OR "foliar endophytes" OR "fruit endophytes". The search period spanned January 1, 2002, to December 31, 2022. A total of 156 valid research articles were collected, excluding non-bibliographic productions, such as conference abstracts, meeting abstracts, editorials, and proceeding papers. The information from these articles was exported in the form of "plain text format" by following the specifications of bibliometric software. To facilitate subsequent processing, plain text files were prefixed with "download_#" with the content "Full Record and Cited References".

### Methodology

CiteSpace is a commonly used bibliometric tool for visualizing knowledge maps, research hotspots, and emerging trends (Singh et al. 2021). In this study, we used CiteSpace v6.1.R6 to visualize and analyze the collected literature data of phyllosphere endophytes. CiteSpace can produce a variety of visualization graphs to display the relationship between different nodes and edges. The different node types include authors, institutions, countries, keywords, articles, cited authors, and cited journals (Li et al. 2019; Singh et al. 2021). In the current study, CiteSpace was employed to analyze and draw the three types of visualized knowledge maps, i.e., co-authorship, co-citation, and keywords occurrence networks for investing the status of phyllosphere endophytes. We analyzed the data from 2002 to 2022 using a time slice of one year, selecting Keyword, Country, and Institution as node types, while retaining other default settings. In parallel, Excel 2010 was used to conduct quantitative analyses to determine the number of publications by country, institution, and year. In this study, the main countries, institutions, evolutionary processes, and frontier hotspots were determined by analyzing measurement results and scientific knowledge graphs.

#### Co-authorship analysis

Over the past few years, the scientific cooperation between different institutes, organizations and countries has increased substantially and gained more attention in recent years as the phyllosphere endophyte research has experienced rapid growth. It is imperative to analyze co-authorship in order to examine and assess researchers' collaborative networks thoroughly. An article with multiple authors signifies that there is a collaborative relationship between these authors, their institutions, and even their countries. In this study, we analyzed the cooperation networks from the perspectives of the authors, countries, and research institutes in the field of phyllosphere endophyte. This study also presents a detailed analysis of the distribution of research power and the intensity of collaboration among different nodes in the global research network. Moreover, the betweenness centrality measure was used in the cooperation network to represent the central position of nodes. A centrality measure includes the degrees of centrality, closeness centrality, or betweenness centrality. This study utilized the betweenness centrality, which measures the number of times an individual node connects to other nodes and demonstrates a node's control over information distribution in a network. A high betweenness node plays a crucial role in connecting the networks and has a pivotal role in the network. Additionally, they facilitate the flow of information across the network. Nodes with low betweenness centrality tend to be less influential in terms of connecting different segments of a network and may not play an important role in facilitating information flow. They may be important within specific clusters, but they are not crucial for connecting them. However, if the value is > 0.1, then nodes could play a central role and have a higher influence.

#### Co-citation analysis

Co-citation is defined as the frequency with which academic publications cite two items from prior literature simultaneously. A co-citation relationship is developed when two or more publications or authors are cited simultaneously in the third article. Co-citation analysis is also used to determine the degree of inter-relationship between authors or articles by establishing mapping connections to represent the research field. In the current study, the journal co-citation network and literature co-citation network were generated using CiteSpace to recognize the influential journals and literature.

#### Keywords co-occurrence analysis

Keywords are typically used to summarize the core content of an article, reflecting the article's value and direction. Using a keywords co-occurrence analysis, we can determine which research topics are hot in a particular field by analyzing the keywords of an article in that field. Hence, co-occurrence network analysis of high-frequency and high-centrality keywords allows researchers to identify emerging trends and hotspots within their field. In this study, CiteSpace was used to construct a keyword network map based on analysis of 156 research papers.

## Data Availability

Not applicable.

## Abbreviations

WoSCC: Web of science core collection
WoS: Web of science
NRPS gene: Non-ribosomal peptide synthetase gene
PKS gene: Polyketide synthetase gene
P NATL ACAD SCI USA: Proceedings of the National Academy of Sciences, USA
